# Supplementary material for: The Effects of Neuromuscular Training on Electromyography, Lower Extremity Kinematics, and Ground Reaction Force During an Unanticipated Side-Cut on Recreational Female Hockey Players
Source: Bioengineering (Basel). 2025 Oct 13;12(10):1101. doi: 10.3390/bioengineering12101101 (PMC12561317; doi:10.3390/bioengineering12101101)
Supplement: Supplementary file 1 [file bioengineering-12-01101-s001.zip › bioengineering-3745446-supplementary.pdf]

The Hockey warm-up

This hockey specific neuromuscular training preparatory warm-up has been developed from the literature to be empirically tested. It has been formatted to conform with the TIDieR guidelines (Hoffman et al., 2014). The equipment required to complete the intervention is cones, hockey sticks, hockey balls, a tape measure, and a stopwatch. The intervention is optimised when delivered by a trained instructor, coach or movement specialist to provide demonstrations and feedback. This intervention should be completed before either training and competition, either on a hockey pitch or other hard, flat surface. Each participant should look to perform all of the exercises; however, if this is not possible, then complete as much of each repetition as possible and complete more the next time.

Part	Exercise	Reps/time/ distance	Technique points and guidance
Part A<br><br>Pulse raiser	Russian walk	5 metres	Walking on heels, straight legs, upright posture. Arms out for balance.
Mobilisation	Jog with plantar flexion	10 m	Jog with upright posture, arms swing as normal, as feet landing dorsi flex so land on balls of feet then heel down and repeat with other leg. Arms out for balance.
Running muscle activation<br><br>Running technique	Foot stamping	5 reps on each side	Hold on to support (player, fence) upright posture, flex standing leg. Active leg, (Phase 1) knee up to parallel to floor knee fully flexed under hips, (Phase 2) full extension, increasingly quickly (Phase 3) extend knee and stamp foot to the floor. Non-support arm out for balance. Reduce or release support arm to increase balance requirements. Focus on reducing/eliminating hip adduction.
	'Ready position'	1	Feet shoulder-width apart, trunk and knees comfortably bent, shoulders back, eyes looking forward. Participants educated on low-risk landing position. Feedback provided.
	Deep squats (or as deep as possible)	10	Flex knees and squat until just past thighs parallel to the floor and butt touch a box if it was there.
	Arabesque	X 3 each leg	Start with hands on hips with support leg on the ground and slight knee flexion. With other that is straight, lift leg up to the back and trunk leans

			forward and bring hands out to the side. Then return to start position. A slow movement.
	Whole running action forwards	X 20 m	Upright posture, knee to parallel during mid-swing, active plantar flexion during foot contact.
(Pivot into)	Jog backwards	X 20 m	Drive backwards.
	Mini skip into gradual increase in distance 2nd time round skip for height	20 m	Hop to mini step and increasingly forward propulsion. Increase arm action to aid larger hop.
	Side shuffle (left leg lead for 10 m then right leg lead for 10 m)	20 m	In crouch position (ready position in hockey) – trunk slightly flexed, knees slightly bend and shuffle sideways.
	Repeat x2 last 4		
Part B<br>Hip mobility	Over hurdle<br>(Trial leg)	X 5	Plant support leg next to ‘hurdle’. Bring active leg back (extend hips) and flex knee. Circumduction of femur. Opposite arm out to the side to reduce trunk twist. Upright stance to reduce anterior and lateral pelvic tilt.
Motor control	Over hurdle backwards	X 5	Plant support leg next to ‘hurdle’. Lift knee up to the front parallel to the floor and raise foot to the side and circumduct femur backwards. Trunk remain upright and opposite arm to activate, out to the side to reduce pelvis /trunk movement.
Posture<br>NMT	Over sideways	X 5	Step over hurdles, when first leg lands, other leg begins action. Support leg is near hurdle. Lead leg lifts vertically to parallel to the ground with knee flexion, then abducts with slight weight transfer and lands on the other side of the hurdle.
	Under hurdle (squat under hurdle)	X 5	A lateral squat action. Bend primarily at the knee with heels on the ground and trunk as upright as possible.

	Repeat with other leg lead		
	Lunge complex— lunge forward, forward diagonal lunge, and backward	1 of each on each side and repeat	Upright posture with hands on hips, lift thigh (as if over a hurdle) step forward, flex knees until the back knee almost touches the ground and return. As a step, lift arms to the side to avoid pelvic tilt.
	Lunge complex with rotation	1 of each on each side and repeat	One of each lunge and first time rotate into bent leg, second time rotate away from bent leg.
Part C Activation	Caterpillar walk	X5	Start in press up position. Walk feet into hands as far as possible – keep back and legs straight and then walk hands out to return to press up position and pause 3 secs.
Mobility/Core activation	Bear walk	10 steps	On all fours with knees off the ground, move opposite hands and feet forward. Hips just above shoulder height (5 movements forward each side).
	Lizard walk	6 steps	On all fours with knees off the ground, move opposite hands and feet forward. Shoulders and hips close to the ground (3 movements forward each side).
	Bear walk unanticipated	10 steps	Shoulder girdle activation, trunk stabilisation rectus abdomanis, erector spinae, internal/external oblique. Leg flexors and extensors used in a more minor way. In pairs, face each other and follow the leader and swap over (5 movements forward each side).
	Lizard walk diagonal	6 steps	On all fours with knees off the ground, move opposite hands and feet forward. Shoulders and hips close to the ground. In pairs, face each other and follow the leader and swap over (3 on each side).

After each primal movement	Crab-like pelvic bridge. On both legs	3	Turnover on to face the sky, on all fours push hips to form a tabletop.
Part D<br><br>Balance	Balance on one leg with ball throw	X10 total	Balance on one leg with leg slightly flexed. Throw hockey ball to partner at a variety of height and sides. Then change legs.
(Straight into)	Hopping on one leg	x10 total	Hopping on one leg with leg slightly flexed. Throw hockey ball to partner at a variety of height and sides. Then change legs.
	Multi directional hopping (figure of 8)	X2 figures on each leg	Start on one leg and hop diagonally left, then laterally right, diagonally backwards left and laterally right. Repeat in other direction and other leg.
	Forward hop and hold	8 on each leg	Hop forward, land using ‘ready’ position and hold, then repeat x 8 and repeat on other leg. Single leg balance.
	Diagonal bound with secure landing	5 steps on each leg, 10 total	Push off with a slightly flexed torso and land on opposite foot, as land slight flex knees. Trunk should remain slightly flexed. Avoid trunk collapsing (i.e., uncontrolled trunk flexion) and internal rotation of the knee.
(Straight into from bounding)	Two foot jump	16 jumps—4 to each place	Double legged jumps with upright posture with a little trunk flexion but keep shoulders back and look forward. Jump forwards, backwards and to each side and back to centre each time.
	Tuck jumps	10	Squat down and jump up and tuck knees into chest, then land into ready position using a soft landing strategy.
	Broad jumps	10	Two-footed forward jump and land. Land without valgus motion and soft landing strategy.

	Single leg squat	5 per leg (alternate with partner)	One player hold stick for partner to hold on to. One leg flex as much as possible while the other is off the ground and out in front. Avoid knee internally rotating.
	Nordic hamstring	3 x 2	Kneel with partner holding ankles. Lean forward slowly until fall to floor. Hands out ready to catch the body. After 3 swap with partner x 2.
Potentiation agility	Double taps	10 m x 2	Slightly faster than above, both feet touch per space. Increase in contraction speed. Upright posture with high knee lift.
	Forward and back (3 x forward and one back)	10 m x 2	High knee lift and upright posture, shoulders back. Move forward over 3 cones and backwards over 1 cone, and repeat going forward x 5.
	Double taps— lateral	10 m x 2	A lateral agility task with both feet touching the ground inside and outside the ‘agility ladder’.
	Hop over	10 m x 2	Hop over each cone 1 leg per 10m.
Part E Potentiation<br><br>Unanticipated move	Sprint 15 m x 3	15 m x 2	Sprint, maximal activity with upright posture. Implementing running technique earlier.
(pivot into)	Sprint backwards	15 m x 2	After sprinting forwards, pivot and sprint backwards in a straight line.<br><br>Similar muscles but a change in emphasis.

	Run and sudden stop	5 m x 2	Coach or partner says stop to a player who is running to develop breaking patterns. Maintain upright trunk and avoid postural sway and uncontrolled trunk flexion.
	Run and cut	15 m x 2 approx.	Diagonal run and change direction, 3 cuts to each side. Avoid femur internal rotation during cutting action.
	Run and cut unanticipated	2 unplanned cuts	Run forwards and cut to a direction given by coach/partner x 2 (does not have to be equal). Biomechanics— flex knee, avoid internal rotation of femur.
	Continuous tig	1 min	A tagger chases another player and touches on the shoulder region to transfer the tag to another player. Previous tagger becomes a player and repeat.

TiDIER Intervention checklist:

1. Name of intervention with brief description.
2. Describe the rationale, theory or goal of the elements essential to the intervention.
3. Materials used—cones, hockey sticks, hockey balls, and stopwatch.
4. Procedures, activities, or processes.
5. Expertise, background, and any specific training.
6. Modes of delivery.
7. Location.
8. When, how much, etc.
9. Tailoring.
